# Tageslichttherapie auf der Intensivstation: eine Übersichtsarbeit

**DOI:** 10.1007/s00063-026-01423-8

**Published:** 2026-03-10

**Authors:** Sarah Lohmeier, Lars Krüger, Thomas Mannebach

**Affiliations:** 1 Intensivstation E 0.1, Herz- und Diabeteszentrum NRW, Universitätsklinikum der Ruhr-Universität Bochum, Georgstraße 11, 32345 Bad Oeynhausen, Deutschland; 2 Stabsstelle Projekt- und Wissensmanagement/Pflegeentwicklung Intensivpflege, Pflegedirektion, Herz- und Diabeteszentrum NRW, Universitätsklinikum der Ruhr-Universität Bochum, Bad Oeynhausen, Deutschland; 3 Stabsstelle Pflegeentwicklung, Pflegedirektion, Herz- und Diabeteszentrum NRW, Universitätsklinikum der Ruhr-Universität Bochum, Bad Oeynhausen, Deutschland

**Keywords:** Chronotherapie, Delirprävention, Evidenzbasierte Pflege, Schlafhygiene, Zirkadianer Rhythmus, Chronotherapy, Delirium prevention, Evidence-based nursing, Sleep hygiene, Circadian rhythm

## Abstract

**Hintergrund:**

Als neuropsychiatrisches Syndrom verschlechtert das Delir die Prognose kritisch kranker Personen auf der Intensivstation (ITS). Risikofaktoren wie Schlafmangel begünstigen die Delirinzidenz. Eine gezielte Anwendung von Licht nimmt Einfluss auf den zirkadianen Rhythmus und Tageslichtinterventionen können zur Regenerierung des natürlichen Tagesrhythmus beitragen. Bisher fehlt es an einer Übersichtsarbeit zur Tageslichttherapie (TLT) für den deutschsprachigen Raum.

**Ziel der Arbeit:**

Erstellung einer Übersichtsarbeit über die aktuell verfügbare Evidenz zum Einsatz der TLT bei kritisch kranken Personen auf der ITS mit den medizinischen Schwerpunkten Kardiologie oder Thorax- und Kardiovaskularchirurgie und mögliche Auswirkungen auf das Delir und den Schlaf.

**Material und Methoden:**

Systematische Literaturrecherche in den Datenbanken CareLit (hpsmedia, Hungen, Deutschland), Cochrane Library (The Cochrane Collaboration, London, U.K.), Livivo (Deutsche Zentralbibliothek für Medizin – Informationszentrum Lebenswissenschaften, Köln, Deutschland) und Medline via PubMed (U.S. National Library of Medicine, Bethesda, MD, USA). Es wurde deutsch- und englischsprachige Literatur eingeschlossen, die kritisch kranke Personen ≥ 18 Jahre im Kontext von Lichttherapie einbezog. Eine kritische Beurteilung erfolgte mit den Beurteilungsbögen von Behrens und Langer.

**Ergebnisse:**

Insgesamt konnten 14 Volltexte eingeschlossen werden, die größtenteils mehrere nichtpharmakologische Interventionen adressierten. Es zeigte sich eine heterogene Studienlage zur Art der Lichtapplikation, Anwendungszeiten und -dauer. Es konnten keine statistisch signifikanten Auswirkungen auf die Delirinzidenz, jedoch positive Effekte im Bereich der Schlafförderung und Psyche aufgezeigt werden.

**Schlussfolgerung:**

Zur TLT auf der ITS fanden sich wenig aussagekräftige Studien. Ein Großteil bezog multikomponente Interventionsstrategien ein. Die vorherrschende Evidenz lässt Rückschlüsse auf positive Wirkungen im Bereich der Schlafförderung und Psyche zu, die im Rahmen einer Präventionsstrategie zur Senkung der Delirinzidenz beitragen könnten. Weitere Forschung ist nötig.

**Zusatzmaterial online:**

Zusätzliche Informationen sind in der Online-Version dieses Artikels (https://doi.org/10.1007/s00063-026-01423-8) enthalten.

## Abstract (en)

**Background:**

As a neuropsychiatric syndrome, delirium worsens the prognosis of critically ill patients in the intensive care unit (ICU). Risk factors such as sleep deprivation increase the incidence of delirium. Targeted light exposure influences the circadian rhythm, and daylight interventions can help to restore the natural daily rhythm. To date, a review of daylight therapy for German-speaking countries is lacking.

**Objective:**

This work aims to provide an overview of the currently available evidence on the use of daylight therapy in critically ill patients on the ICU, with a medical focus on cardiology or thoracic and cardiovascular surgery. The potential effects of daylight therapy on delirium and sleep are discussed.

**Materials and methods:**

A systematic literature search was conducted in the databases CareLit (hpsmedia, Hungen, Germany), Cochrane Library (The Cochrane Collaboration, London, U.K.), Livivo (German National Library of Medicine – Information Centre for Life Science, Cologne, Germany), and Medline via PubMed (U.S. National Library of Medicine, Bethesda, MD, USA). German- and English-language literature involving ICU patients at least 18 years of age in the context of light therapy was included. A critical appraisal was performed using the assessment tools developed by Behrens and Langer.

**Results:**

Fourteen full texts were included, most of which involved multiple nonpharmacological interventions. The studies varied in terms of light application mode, timing, and duration. No significant effects on the delirium incidence were found, but positive effects on sleep promotion and psychological outcomes were observed.

**Conclusion:**

There are few conclusive studies on daylight therapy in the ICU. Most involved multicomponent intervention strategies. The prevailing evidence suggests beneficial effects on sleep promotion and psychological outcomes, which could contribute to delirium prevention strategies. Further research is needed.

## Einleitung

Störungen des natürlichen Tag-Nacht-Zyklus scheinen einen Einfluss bei der Entwicklung eines Delirs zu haben [17]. Tageslicht ist u. a. entscheidend für einen natürlichen Schlaf- und Wachrhythmus. Auf der Intensivstation (ITS) mangelt es jedoch häufig an natürlichem Tageslicht. Geräte zur Simulation von Tageslicht könnten einen Beitrag zur Schlafförderung, Rückgewinnung eines normalen Tag-Nacht-Zyklus und somit zur Delirprävention leisten [36]. Diese Arbeit soll eine Übersicht über die aktuell verfügbare Evidenz zur Tageslichttherapie (TLT) auf der ITS geben.

## Hintergrund

Es ist lange bekannt, dass Licht einen primären Synchronisationsreiz für den zirkadianen Rhythmus darstellt [6]. Der menschliche Körper ist an einen 24-Stunden-Zyklus gebunden, wobei sich zahlreiche Körper- und Verhaltensrhythmen an die Außenwelt koppeln. Dieser Vorgang wird auch Chronobiologie genannt. So entsteht ein natürlicher Tagesrhythmus [33]. Der zirkadiane Rhythmus umschreibt explizit die Physiologie des Schlaf-Wach-Rhythmus [32]. Kritisch kranke Personen in komplexen, multidisziplinären Versorgungssituationen – etwa auf der ITS – sind zahlreichen Störfaktoren und medizinischem Equipment ausgesetzt [3]. Externe Faktoren wie künstliches Licht können Schlaflosigkeit, zirkadiane Störungen und Delir begünstigen. Gleichzeitig kann Licht aber auch als gezielte Intervention eingesetzt werden [3, 8].

Das Delir gehört zu den sog. Bewusstseinsstörungen. Dabei ist es keine eigenständige Krankheitsentität, sondern eine Folge medizinischer Faktoren. Es umfasst einen akuten, fluktuierenden, aber in der Regel reversiblen Zustand einer zerebralen Dysfunktion. Das Delir ist durch akute Veränderungen der Aufmerksamkeit, des Bewusstseins und der Kognition gekennzeichnet [39]. Dies verursacht eine schlechtere Prognose und steht in Verbindung mit einer längeren Beatmungs‑, Intensiv- und Krankenhausaufenthaltsdauer [17]. Diverse Risikofaktoren für ein Delir wurden innerhalb der Intensivtherapie identifiziert. Dazu gehören beispielsweise Erkrankungen, wie die Herzinsuffizienz oder Sepsis, aber auch das Alter, unbehandelte Schmerzen und Schlafmangel [2]. Lindroth et al. [18] berichteten im Rahmen einer weltweiten Punktprävalenzstudie zum „world delirium awareness day“ am 15. März 2023 eine Delirprävalenz von rund 20 % auf ITS, *high acuity* und *intermediate medical car*e Stationen. In Deutschland, Österreich und der Schweiz zeigten sich ähnliche Ergebnisse [12].

Abgesehen vom erhöhten Delirrisiko ist Schlafmangel für viele kritisch kranke Personen eine häufige und belastende Beschwerde. Diese Schlafstörungen können ausgeprägt sein und äußern sich durch Schlaffragmentierung, abnorme zirkadiane Rhythmen, vermehrten Leicht-, verminderten Tief- und Rapid-Eye-Movement (REM)-Schlaf [9]. Bereits gesunde Personen zeigen bei solchen Schlafstörungen eine verminderte neurokognitive Leistungsfähigkeit und Gedächtniskonsolidierung. Kritisch kranke Personen benötigen Hilfe zur Kompensation [28].

**Abb. 1:**
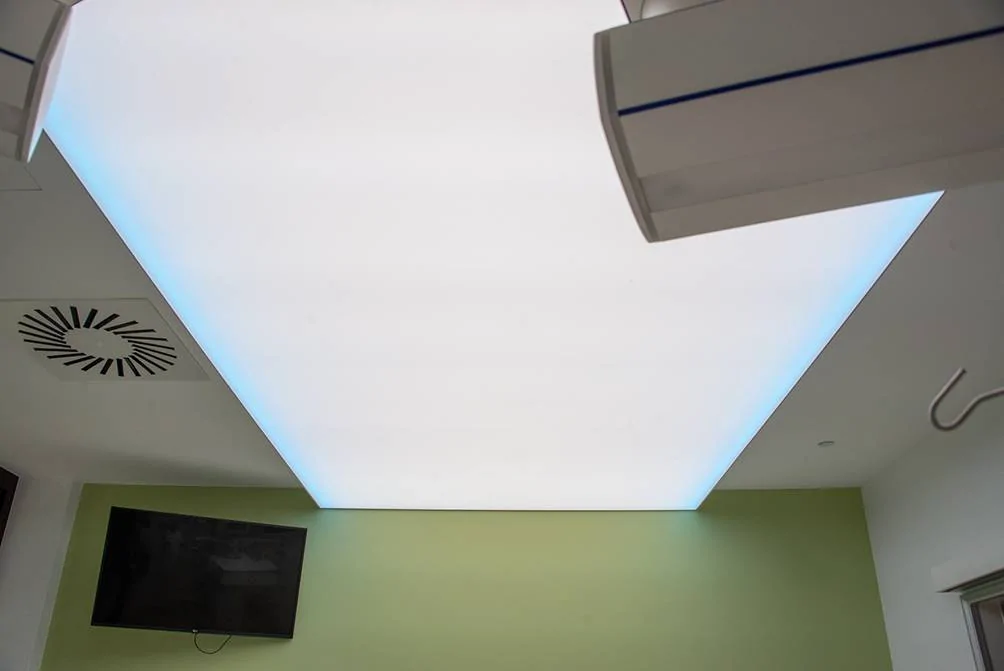
Tageslichtsimulierendes Device (TLD) an der Zimmerdecke auf einer Intensivstation (ITS). Dynamische Lichtinstallationen ermöglichen den kritisch kranken Personen, auf Zimmerebene aktiv zu sein, während eine statische Lösung sie oft dazu zwingt, in Position zu bleiben. Diese TLD sind kostenintensiv und erfordern Umbaumaßnahmen

Licht, besonders im Blaulichtspektrum, kann Wachheitsreaktionen hervorrufen und somit gezielt angewendet werden [8]. Es existieren verschiedene tageslichtsimulierende Devices (TLD) für den Einsatz auf der ITS (Abb. 1 und 2). Diese reichen von einfachen Leuchtkästen mit hellem Licht einer bestimmten Wellenlänge und Intensität bis zu dynamischen Tageslichtbögen, welche die Farbe und Lichtintensität im Laufe des Tags anpassen [19].

**Abb. 2:**
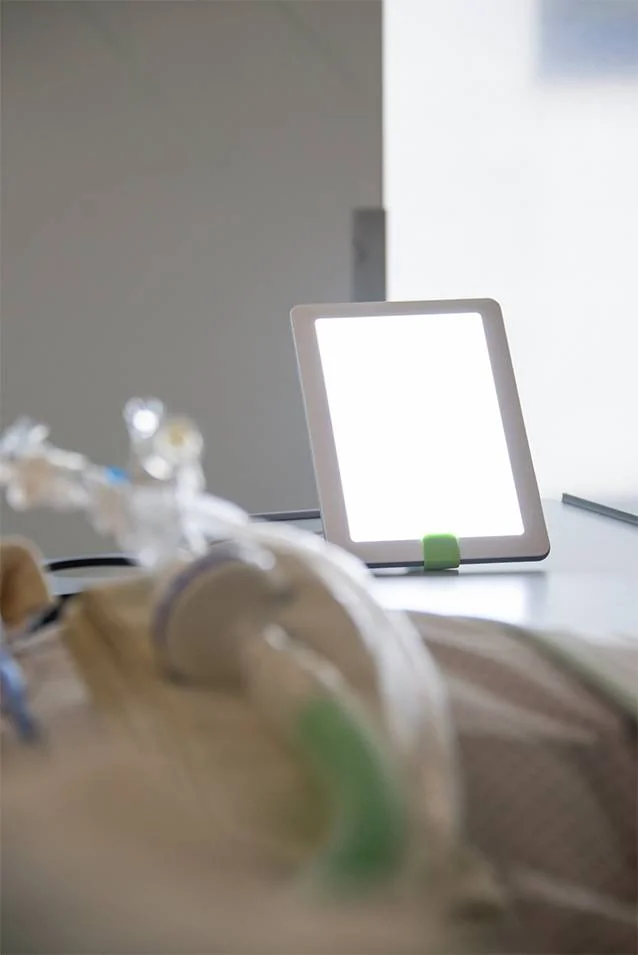
Tageslichtsimulierendes Device (TLD) auf dem Nachtschrank einer Intensivstation (ITS). Es handelt sich um ein Gerät in Größe eines Tablets, das individuell platziert werden kann und manuell gestartet und gestoppt werden muss. Weder die Lichtstärke noch das Farbspektrum können variiert werden. Die Anschaffungskosten variieren je nach Device, sind jedoch preiswerter als z. B. eine Deckeninstallation

Bisher fehlt eine deutschsprachige Übersichtsarbeit, die den aktuellen Forschungsstand zur Tageslichttherapie zusammenfasst.

## Ziel

Ziel dieser Übersichtsarbeit ist es, die aktuell verfügbare Evidenz zum Einsatz von TLD und mögliche Auswirkungen auf das Delir und den Schlaf für kritisch kranke Personen auf der ITS mit den medizinischen Schwerpunkten Kardiologie oder Thorax- und Kardiovaskularchirurgie darzustellen.

## Methodik

Die Berichterstattung dieser Übersichtsarbeit folgt den Empfehlungen der Preferred Reporting Items for Systematic reviews and Meta-Analyses (PRISMA) Extension for Scoping Reviews (PRISMA-ScR; Supplement ESM 1; [35]).

Die Entwicklung einer Recherchestrategie nach dem Patient/Problem, Intervention, Kontrolle/Kontrollintervention und Ergebnis (PIKE)-Schema [4] ergab eine Kombination von Suchbegriffen, die mit logischen Operatoren („and/or“) verknüpft wurden (Supplement ESM 2).

Es erfolgte eine systematische Literaturrecherche im Zeitraum November 2023 bis Juni 2024 sowie eine Nachrecherche Ende Juni 2025 in den Datenbanken CareLit (hpsmedia, Hungen, Deutschland), Cochrane Library (The Cochrane Collaboration, London, U.K.), Livivo (Deutsche Zentralbibliothek für Medizin - Informationszentrum Lebenswissenschaften, Köln, Deutschland) und Medline via PubMed (U.S. National Library of Medicine, Bethesda, MD, USA).

**Abb. 3:**
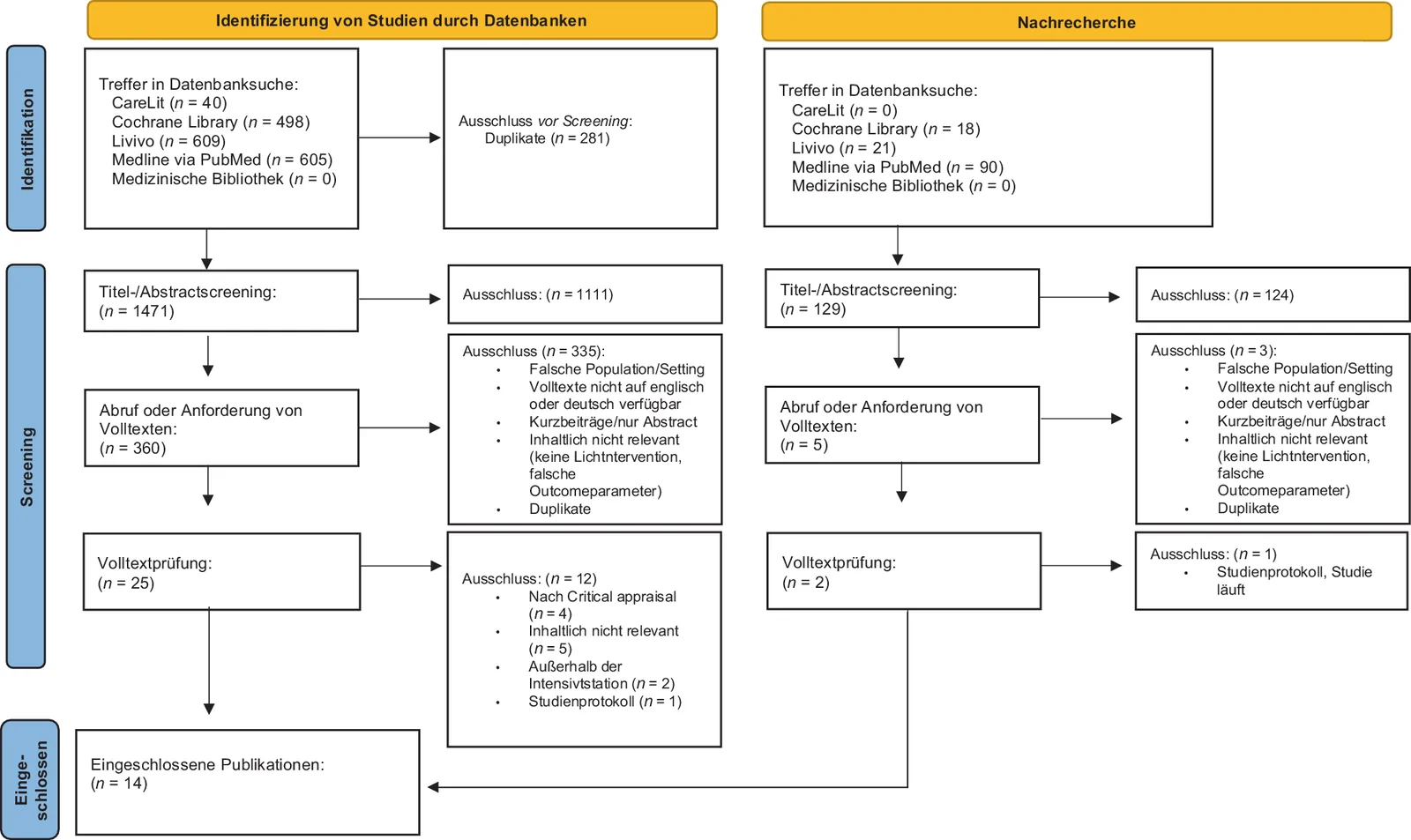
Eigene Darstellung des Flussdiagramms zur Literaturrecherche und -auswahl. (In Anlehnung an Page et al. [26])

Eingeschlossen wurden deutsch- oder englischsprachige Studien, die nicht älter als 20 Jahre waren, kritisch kranke Personen ≥ 18 Jahren einbezogen, die idealerweise auf einer kardio-, thoraxchirurgischen oder kardiologischen ITS behandelt wurden, und Tageslichttherapien in Form von TLD und natürlichem Licht anwendeten. Die Einschränkung auf die letzten 20 Jahre erfolgte aufgrund relevanter Technologien im Rahmen der Behandlungsmethoden wie beispielweise der präziseren Steuerung von LED-Beleuchtungssystemen, der Fokussierung auf aktuelle Evidenz und dem Management der Datenmenge. Nichtrelevante Publikationen wurden zunächst durch ein Titel- und Abstractscreening ausgeschlossen. Ergänzend wurde anhand der Literaturverzeichnisse weitere Literatur nach dem Schneeballprinzip recherchiert. Zusätzlich erfolgte eine Handsuche in der medizinischen Bibliothek des Herz- und Diabeteszentrums NRW (Abb. 3)

Nach Abstract- und Volltextsichtung durch 2 Personen *(SL, TM)* erfolgte eine kritische Beurteilung mit den Beurteilungsbögen von Behrens und Langer [4]. Dabei wurden je nach Qualität und Relevanz nach den jeweiligen Prüfschritten „Schulnoten“ zwischen 1 (sehr gut) und 6 (ungenügend) vergeben.

Die Ergebnisse eingeschlossener Studien wurden einander tabellarisch gegenübergestellt (Supplement ESM 3) und darüber hinaus detailliert (Supplement ESM 4) nach den Themen Delir, Schlaf/zirkadianer Rhythmus, sonstige Ergebnisse, Anwendungshinweise und Kritik zusammengefasst.

Bei Unstimmigkeiten oder Fragen wurde sich an eine 3. Person mit Masterabschluss in Pflegewissenschaft gewendet *(LK*; Abb. 3).

## Ergebnisse

Insgesamt wurden 14 Publikationen nach einer erfolgten kritischen Beurteilung mit einer Bewertung von ≤ 3 eingeschlossen. Das Spektrum der Publikationen umfasste Metaanalysen (*n* = 2; [16, 34]), systematische Übersichtsarbeiten (*n* = 6; [3, 13, 15, 19, 21, 38]); randomisierte Kontrollstudien (*n* = 4; [11, 25, 27, 29]) und prospektive Kohortenstudien/Pilotstudien (*n* = 2; [5, 30]). Mehrere der eingeschlossenen Publikationen bezogen unterschiedliche nichtpharmakologische Interventionen (z. B. Schlafbrillen, Ohrstöpsel, Musiktherapie etc.) ein [13, 15, 16, 21, 27, 34, 38]. Wenige Arbeiten bezogen sich ausschließlich auf die Lichtintervention.

Es zeigt sich eine heterogene Studienlage zur Art der Anwendung von Licht. Diese Interventionen reichten vom Zugang zu natürlichem Tageslicht über Smartphone-Apps bis zu dynamischen Tageslichtbögen. Anwendungszeiten und -dauer unterschieden sich ebenso. Die TLT mit Tageslichtlampen oder dynamischen Tageslichtbögen (mit unterschiedlichen Luxstärken) erzielten mehrere positive Effekte sowohl bezogen auf die Schlafförderung als auch auf psychologische Parameter [11, 16, 19, 27].

### Senkung der Delirinzidenz

Das Einsetzen von TLD allein zeigte im Großteil der Publikationen keine statistisch signifikante Wirkung auf die Delirinzidenz [3, 5, 13, 19, 21, 25, 29, 38]. Dies spiegelte sich unter anderem in der Studie von Simons et al. [29] wider: Ein Delir trat bei 137 (38 %) von 361 kritisch kranken Personen in der Dynamic-light-application(DLA)-Gruppe und bei 123 (33 %) von 373 kritisch kranken Personen in der Kontrollgruppe auf (Odds Ratio [OR] 1,24; 95 %-Konfidenzintervall [95 %-KI] 0,92–1,68, *p* = 0,16).

Erwies sich ein positiver Effekt in der Inzidenz, aber auch in der Schwere des Delirs, war die Studiengröße gering. So haben beispielsweise in einer Pilotstudie 76 % (*n* = 28) der Studienteilnehmenden in Standardzimmern ein Delir entwickelt, verglichen mit 46 % (*n* = 17) in modifizierten Zimmern mit Lichttherapie (*p* = 0,017; [30]). Die Anwendung von TLD kann im Rahmen einer multikomponenten Präventionsstrategie eingebunden werden [13, 16, 27, 34, 38].

### Schlafförderung

Es gab heterogene Methoden zur Untersuchung der Schlafparameter und der zirkadianen Rhythmik. Es wurde die nichtinvasive Überwachung von Ruhe und Aktivität (Aktigraphie) als Messinstrument mithilfe von unterschiedlichen Gerätschaften eingesetzt. Eine zusätzliche Beurteilung des Schlafs erfolgte durch Pflegefachpersonen, Fragebögen zur Selbsteinschätzung, Schlaftagebücher etc. Nur selten wurde die Polysomnographie verwendet [11, 16, 19, 21, 25]. In der Übersichtsarbeit von Lindskov et al. [19] werden beispielsweise zur Beurteilung des Schlafs > 15 Messinstrumente aufgelistet. Zusätzlich wurden unterschiedliche Begrifflichkeiten genutzt. Die Ergebnisse sind je nach Design, Population und Ergebnismessung heterogen.

Der Großteil der identifizierten Literatur konnte einen Anstieg der Schlafdauer und -qualität ausmachen. Dies zeigte sich beispielsweise durch eine verringerte Latenzzeit des Schlafbeginns, weniger nächtliches Erwachen, tiefere Schlafstadien und einen Rückgang der nächtlichen Unruhe. Ono et al. [25] legte dies durch niedrigere Aktivitätswerte und statistisch signifikante Unterschiede zwischen den Interventionsgruppen um 2.00, 4.00, 6.00 und 7.00 Uhr dar (*p* < 0,05; [19, 21, 25, 27, 34]). Spies et al. [30] berichteten hierzu in einer Pilotstudie von einer Erhöhung des Melatoninwerts (*p* = 0,0001). Andere Studien zeigten keine Veränderung in der selbstberichteten Schlafqualität [11, 15].

### Sonstige Effekte

Die Studien zu TLD belegen Wirkungen im Bereich des Schlafs, aber auch im Rahmen der Kognition und Stimmung [11, 19]. So wurde in mehreren qualitativen Studien festgehalten, dass angemessene Beleuchtungsniveaus tagsüber Ruhe und Sicherheit vermitteln. Das Licht wurde als angenehm und ebenso stimmungsaufhellend empfunden [11, 19, 21]. Zusätzlich wurde ein erhöhter Wachzustand beschrieben [21].

### Anwendungshinweise

Die künstliche Lichtquelle sollte an den natürlichen Tag‑/Nachtrhythmus angelehnt werden. Dabei wird eine Anwendungsdauer von 1–5 h beschrieben, die sich auf den Morgen und die Mittagszeit verteilten [11, 19, 25, 27, 29]. Bei einer Anwendung von TLD ≤ 30 Min erwiesen sich keine Veränderungen hinsichtlich der Stimmung und der Kognition. Eine positive Auswirkung ergab sich erst ab einer 2‑stündigen Anwendung pro Tag [19]. Lindskov et al. [19] fassten zusammen, dass nur Interventionen, die > 5 Tage andauerten eine Wirkung zeigten.

Die Lichtquelle sollte dabei möglichst im Blickfeld der kritisch kranken Personen stehen. Hierbei werden unterschiedliche Abstände erprobt (30–140 cm), die von der Luxstärke und Dauer der Anwendung festgemacht werden [19]. Ebenso empfiehlt sich eine Oberkörperhochpositionierung von 30–45° [25, 27]. Kritisch kranke Personen, die TLT zur Anpassung des zirkadianen Rhythmus erhalten, sollten möglichst wach sein, da so kurzwelliges blaues Licht über die Netzhaut aufgenommen werden kann. Im Fall von geschlossenen Augenlidern ist der Effekt entsprechend geringer [19, 38]. Während der Lichtexposition ist es notwendig, dass das betreuende medizinische Personal (Pflegefachpersonen oder ärztlicher Dienst) in der Nähe der kritisch kranken Person bleiben und diese klinisch beobachten, da sie die Therapie häufig nicht selbst beenden können. Hierzu können erweiterte Assessments, wie beispielsweise die numerische Ratingskala (NRS) für Angst- und Schmerz, eingesetzt [1] oder klinische Anzeichen von Stress (z. B. Tachykardie, Agitation etc.) als Indikation für eine Beendigung der Maßnahme beobachtet werden [2]. Die Hauptergebnisse wurden in Infoboxen (Supplement ESM 4) zusammengefasst.

### Unerwünschte Nebenwirkungen/Kontraindikationen

Die TLD sollten nicht bei Erkrankungen der Augen, wie Entzündungen, Glaukom, diabetische Nephropathie und sonstigen Erkrankungen des Sehnervs, angewendet werden. Die Anwendung von TLD ist bei Menschen mit ausgeprägter Lichtempfindlichkeit bzw. Migräneanfälligkeit kontraindiziert bzw. bedarf der ärztlichen Absprache, da Photophobie und Kopfschmerzen als Nebenwirkungen auftraten [29]. Devices mit einer Luxstärke von über 10.000 lx wurden als zu exzessiv beschrieben [38]. In manchen Fällen wurde eine erhöhte Augenbelastung bzw. -ermüdung dargestellt [19]. Zusätzlich können bei kleinen TLD mit einer starken Luxstärke die Blendeffekte verstärkt werden und möglicherweise zum Auftreten eines Delirs beitragen [30]. Im Allgemeinen gilt Licht aber als gut verträglich und wird als nichtinvasive Intervention angesehen [19].

## Diskussion

Ziel dieser Übersichtsarbeit war es, die Evidenz für den Einsatz von TLD auf kardiologischen oder thorax- und kardiovaskularchirurgischen ITS und die Auswirkungen auf das Delir und den Schlaf aufzuzeigen. Es gibt viele Forschungsberichte, die sich auf kombinierte schlaffördernde Maßnahmen beziehen [3, 5, 13, 16, 21, 27, 34, 38], jedoch eine begrenzte Menge an Primärforschung im Rahmen der Lichttherapie mit primärem Outcome der Delirinzidenz auf der ITS [11, 19, 25, 29, 30]. Nach der kritischen Bewertung wurden heterogene Ergebnisse für die Wirksamkeit der Interventionen identifiziert.

Die Wirkung von TLD auf die Vorbeugung oder Behandlung des Delirs war in den eingeschlossenen Studien heterogen, doch scheint sie mit der Lichtintensität und der Dauer der Intervention zusammenzuhängen. Parallel ist unklar, welche Auswirkungen diese im Kontext von weiteren Maßnahmen zur Delirprävention haben. So berichteten z. B. Exl et al. [12] von vielschichtigen Maßnahmen zur Delirprävention im deutschsprachigen Raum, bei denen die TLT allerdings nicht speziell aufgeführt wird, jedoch die Schlafförderung.

Insgesamt wurden der TLT positive Eigenschaften nachgewiesen, die Vorgehensweise und die eingesetzten TLD unterschieden sich allerdings in den Studien. Dabei wird in den meisten Arbeiten eine multikomponente Interventionsstrategie empfohlen [3, 5, 15, 16, 21, 27, 29, 31, 34]. Dies gilt ebenso für das Ergebnismaß der Schlafförderung. So wird zwar in einigen Studien eine bessere Schlafqualität beschrieben [16, 19, 21, 25], dennoch waren die Messinstrumente zur Feststellung dieser sehr unterschiedlich [11, 21, 25]. Im Rahmen eines Selbsteinschätzungsinstruments ist ein Placeboeffekt nicht auszuschließen. Ähnliches gilt für die nichtverblindete Bewertung der Studiengruppen und somit eine mögliche Entstehung eines Beobachter- bzw. Bewertungsbias [11]. Der Goldstandard der Erhebung von Schlafstörungen wäre die Polysomnographie [22], die biophysiologische Veränderungen aufzeichnet (Muskel‑, Gehirn und Augenaktivität). Die Messmethode fand allerdings kaum Anwendung. Dies scheint den erschwerten Umständen der Umsetzung auf der ITS anzulasten zu sein.

Gezielte Lichtexposition zeigt in den eingeschlossenen Studien einen Einfluss auf den zirkadianen Rhythmus [3, 16, 19, 25, 30], die Auswirkung auf Schlaf und Delir bei kritisch kranken Personen auf der ITS ist jedoch noch nicht ausreichend erforscht. Bei der Interpretation der Wirksamkeit einer multikomponenten Strategie sollte berücksichtigt werden, dass der beobachtete Effekt nicht eindeutig einzelnen Komponenten zugeschrieben werden kann. Es besteht die Möglichkeit von Confounding-Effekten, bei denen sich die einzelnen Maßnahmen gegenseitig beeinflussen oder der Gesamteffekt durch eine nichtkontrollierte Variable erklärt werden könnte [37].

Die jeweils angewandten Geräte, Interventionsdauern und Luxniveaus variierten in den eingeschlossenen Studien beispielsweise von Smartphone-Apps [15] über Tageslichtlampen [21, 25, 27] bis hin zu dynamische Tageslichtbögen [21, 29, 30]. Die Auswirkungen bei niedrigeren Luxwerten variierten stärker als in Studien mit höheren Werten. Einige Studien mit hoher Beleuchtungsstärke zeigten jedoch keine besseren Effekte. Somit scheint die reine Luxstärke selbst nicht proportional zum Ergebnis zu sein. Studien zur zirkadianen Verzögerung zeigen positive Ergebnisse sowohl bei längeren als auch bei kürzeren Interventionen [19].

Nach Forschungsergebnissen zu den Melanopsinganglienzellen der Netzhaut ist nun bekannt, dass nicht nur die Lichtintensität, sondern vielmehr die Verteilung der Wellenlängen, insbesondere des kurzwelligen blauen Lichts, den Nucleus suprachiasmaticus beeinflussen [23]. Nur wenige der eingeschlossenen Studien haben über die Verteilung des Lichtspektrums berichtet, sodass die Luxzahl nur ein Näherungswert sein könnte, da eine allgemeine Erhöhung der Luxzahl die Intensität der einzelnen Wellenlängen erhöht [20]. Dementsprechend existiert derzeit keine standardisierte Anwendung von Licht.

Zudem können andere Faktoren einer intensivmedizinischen Behandlung, wie Lärm, mechanische Beatmung und fehlende soziale Anreize, die Expression von Chronobiologie beeinflussen und somit die Wirkung von Licht auf den zirkadianen Rhythmus hemmen [19]. Ebenso nehmen krankheitsbedingte Faktoren wie beispielsweise die Sepsis Einfluss auf die zirkadiane Melatoninsekretion [24]. So wird unter anderem empfohlen, Studien an einer nicht intensivmedizinischen Population durchzuführen, um die zirkadiane Regulation ausreichend für eine Standardisierung bewerten zu können [19] oder Pilotprogramme/-räume auf der ITS zu nutzen.

Die Installation von dynamischen Tageslichtbögen auf der ITS gilt derzeit noch als wirtschaftliches Risiko, da die Evidenz noch überschaubar ist. Jedoch steht fest, dass das Lichtniveau tagsüber in Behandlungszimmern von kritisch erkrankten Personen zu niedrig ist und es häufig zu nächtlichen Lichtexpositionen kommt [10], z. B. durch medizinisches Personal. Die Einsparungen könnten jedoch potenziell hoch sein, wenn z. B. durch eine gezielte Anwendung eine Verkürzung der Aufenthaltsdauer auf der ITS erfolgen würde. Alternativ könnten tragbare TLD genutzt werden (Abb. 2). In jedem Fall bedarf es einer Schulung des Personals.

## Stärken und Limitationen

Eine Stärke dieser Arbeit ist, dass eine systematische Literaturrecherche in 4 wissenschaftlichen Datenbanken durchgeführt wurde. Die kritische Beurteilung der eingeschlossenen Volltexte erfolgte mit den Beurteilungschecklisten von Behrens und Langer, die international weniger zum Einsatz kommen, jedoch national viel Berücksichtigung in der Hochschullehre finden [14] und vom *German Center for Evidence-based Nursing* zur Verfügung gestellt werden.

Die Aussagekraft dieser Übersichtsarbeit hat Limitationen. Es besteht die Möglichkeit, dass geeignete Studien übersehen wurden, da das Erscheinungsjahr der eingeschlossenen Publikationen zur Eingrenzung der Ergebnisse auf 20 Jahre beschränkt wurde. Der Recherchestrang beinhaltete kardiologische sowie kardio- und thoraxchirurgischen Disziplinen und das Titel‑/Abstract- und Volltextscreening erfolgte nicht verblindet, aber unabhängig voneinander. Dadurch kann es zu einem Reviewerbias gekommen sein.

Zudem weisen die aufgenommenen Studien unsichere Parameter für den Vergleich auf. Es wurde insgesamt eine Vielzahl unterschiedlicher Maßnahmen zur Schlafförderung und/oder Delirprävention bewertet, nur wenige Studien beschäftigten sich ausschließlich mit TLT. Die Studien waren in Bezug auf Design, Setting, Messungen und Dauer der Intervention heterogen.

## Schlussfolgerung

Die Anwendung von TLD auf der ITS rückt zunehmend in den Fokus. Trotz der derzeit heterogenen Studienlage erzielten TLD mehrere positive Ergebnisse. Sie trugen zur Ruhe und Sicherheit bei [11], senkten nächtliche Unruhe [27], förderten den Schlaf und wirkten stimmungsaufhellend [19]. Die Ergebnisse in Bezug auf die Delirinzidenz sind unterschiedlich, variierten mit den Luxstärken und zeigten bei einer isolierten Anwendung keine statistisch signifikante Wirkung. In einem Großteil der Studien empfahl sich eine multikomponente Interventionsstrategie. [3, 5, 15, 16, 21, 27, 29, 31, 34]. Die Komplexität und Multifaktorialität des Delirs in der Intensivmedizin lässt allerdings vermuten, dass keine einzelne Intervention, sondern ein Behandlungsbündel entscheidend zur Senkung der Inzidenz beiträgt [7]. Zukünftig ist es durchaus sinnvoll, eine interdisziplinäre Suchstrategie zu formulieren.

Der genaue Einfluss von TLT auf Delir und Schlaf ist bei kritisch kranken Personen somit noch nicht ausreichend erforscht und andere behandlungsassoziierte Faktoren, wie mechanische Beatmung und Medikamente, beeinflussen ebenso die Chronobiologie. Die TLT ist allerdings ein wichtiger Ansatzpunkt, um die mangelnde Exposition von natürlichem Tageslicht zu kompensieren. Weitere, größere und gut konzipierten Studien sind erforderlich, um diese Ergebnisse zu bestätigen und den Einsatz der Lichttherapie im Setting der Intensivstation zu optimieren.

## Fazit für die Praxis

- Die TLT kann als Teil einer multikomponenten Interventionsstrategie zur Verbesserung des zirkadianen Rhythmus genutzt werden.
- Die Förderung des Tag-Nacht-Rhythmus gilt als präventive Maßnahme für ein Delir.
- Die Intervention sollte individuell auf den/die kritisch kranke Person abgestimmt sein.
- Der Einsatz der TLT sollte in einem Zeitraum von 8.00 bis 15.00 Uhr stattfinden.
- Tragbare TLD sind kostengünstige Alternativen zu dynamischen Tageslichtbögen.

## Supplementary Information
