# Supplementary material for: Daylight therapy in intensive care units: a review
Source: Med Klin Intensivmed Notfmed. 2026 Mar 10;121(6):499–505. [Article in German] doi: 10.1007/s00063-026-01423-8 (PMC13538152; doi:10.1007/s00063-026-01423-8)
Supplement: Supplementary file 2 [file 63_2026_1423_MOESM4_ESM.docx]

**SUPPLEMENT ESM 4**

**Titel**

Tageslichttherapie auf der Intensivstation: Eine Übersichtsarbeit

**Autorenschaft**

*(verblindet)*

**Kasten 1: Anwendungshinweise Tageslichttherapie**

- *Durchführung zu den Zeiten eines natürlichen Lichtpeaks: 8 – 15 Uhr [1, 2, 5, 7, 8]*
- *Anwendungszeit mindestens eine Stunde pro Tag [1, 2, 5, 7, 8]*
- *Muss nicht kontinuierlich erfolgen [1, 2, 5, 7, 8]*
- *Anwendung darf sich über den Morgen und Mittag verteilen [5]*
- *Patient*Innen sollten bei der Anwendung von Tageslicht wach sein*
- *Bei tragbaren Devices: Oberkörperhochlagerung 30 – 45° [5, 7]*
- *Tragbare TLD sollte im Blickfeld der zu behandelnden Person stehen [2]*
- *Abstand mindestens 30cm, maximal 140cm: ist an die Luxstärke anzupassen [2]*
- *Kontraindikationen: Bei Epilepsie, Licht- und Migräneanfälligkeit. Letzteres bedarf ärztlicher
   Absprache [2, 8]*
- *In Patienten*Innen-Nähe bleiben*

**Kasten 2: Unterschied Lux und Lumen**

*Lux (lx), oder auch Beleuchtungsstärke, gibt die Lichtleistung in einem bestimmten Bereich an*

*Lumen (lm), die Maßeinheit für Lichtstrom, gibt an wie viel Licht abgegeben wird.*

*Somit gibt Lux an wie hell die Oberfläche in Lumen pro Quadratmeter (lm/m²) beleuchtet wird und Lumen ist die Einheit für die Lichtmenge, welche eine Lichtquelle pro Zeiteinheit abgibt [4].*

**Kasten 3: Lichtverhältnisse Alltag vs. Intensivstation**

*Kurzwelliges blaues Licht bezeichnet den energiereichen Anteil des sichtbaren Spektrums mit Wellenlängen von etwa 380–500 nm. Wegen der kurzen Wellenlänge enthält es mehr Energie als längerwellige Strahlen und wird auch als High-Energy Visible Light (HEV-Licht) bezeichnet [3].*

*Helles Tageslicht liefert 10 000 Lux, während Dämmerung und Mondlicht 10 bzw. weniger als 0,1 Lux liefern [6].*

*Tagsüber sind Intensivstationen schwach beleuchtet, in der Regel zwischen 100 und 300 Lux [6].*

*Künstliches nächtliches Licht kann Melatonin bei 40 Lux innerhalb von nur 5 Minuten unterdrücken [6].*

**Literatur**

1.	Engwall M, Fridh I, Johansson L et al. (2015) Lighting, sleep and circadian rhythm: An intervention study in the intensive care unit. Intensive Crit. Care Nurs. 31:325-335

2.	Lindskov FO, Iversen HK, West AS (2022) Clinical outcomes of light therapy in hospitalized patients - A systematic review. Chronobiol. Int. 39:299-310

3.	Lucas RJ, Peirson SN, Berson DM et al. (2014) Measuring and using light in the melanopsin age. Trends Neurosci. 37:1-9

4.	Newell DB, Tiesinga E (eds) (2019) The International System of Units (SI). United States version of the English text of the ninth edition (2019) of the International Bureau of Weights and Measures publication. Le Système International d’ Unités (SI).

5.	Ono H, Taguchi T, Kido Y et al. (2011) The usefulness of bright light therapy for patients after oesophagectomy. Intensive Crit. Care Nurs. 27:158-166

6.	Perry H, Alight A, Wilcox ME (2024) Light, sleep and circadian rhythm in critical illness. Curr. Opin. Crit. Care 30:283-289

7.	Potharajaroen S, Tangwongchai S, Tayjasanant T et al. (2018) Bright light and oxygen therapies decrease delirium risk in critically ill surgical patients by targeting sleep and acid-base disturbances. Psychiatry Res. 261:21-27

8.	Simons KS, Laheij RJ, Van Den Boogaard M et al. (2016) Dynamic light application therapy to reduce the incidence and duration of delirium in intensive-care patients: a randomised controlled trial. Lancet Respir Med 4:194-202
